# Yellow Fever: Its Transmission by the Culex Mosquito

**Published:** 1886-11

**Authors:** 


### *YELLOW FEVER: ITS TRANSMISSION BY THE CULEX MOSQUITO.*

Dr. Charles Finlay, of Havana, maintains, in an article which appears in the October issue of *The American Journal of the Medical Sciences*, that yellow fever is not spontaneously transmissible by infection through the air by contact, but that it may be artificially communicated by inoculation, and only becomes epidemic when such inoculations can be verified by some external natural agent, such as the mosquito.

The history and etiology of yellow fever exclude from our consideration, as possible agents of transmission, other blood-sucking insects, such as fleas, etc., the habits and geographical distribution of which in no wise agree with the course of that disease; whereas, a careful study of the habits and natural history of the mosquito shows a remarkable agreement with the circumstances that favor or impede the transmission of yellow fever. So far as Dr. Finlay's information goes, this disease appears incapable of propagation wherever tropical mosquitos do not or are not likely to exist, ceasing to be epidemic at the same limits of temperature and altitude which are incompatible with the functional activity of those insects; while, on the other hand, it spreads rapidly wherever they abound. From these considerations, taken in connection with his successful attempts in producing experimental yellow fever by means of the mosquito's sting, it is to be inferred that these insects are the habitual agents of its transmission. It cannot be denied, however, that other such agents may and probably do occasionally occur, but not being endowed with the same facilities for rapid and extensive operation, their influence becomes insignificant with the action of the Cuban culex.

